# Supplementary material for: Knowledge syntheses in medical education: A bibliometric analysis
Source: Perspect Med Educ. 2020 Oct 22;10(2):79–87. doi: 10.1007/s40037-020-00626-9 (PMC7580500; doi:10.1007/s40037-020-00626-9)
Supplement: Supplementary file 1 — Appendix: Database search strategies [file 40037_2020_626_MOESM1_ESM.docx]

Appendix: Search Strings

**PubMed Search String (LIMIT: 1999/01/01 – 2019/12/31)**

("knowledge synthesis" [title/abstract] OR "literature review"[title/abstract] OR  "evidence synthesis" [title/abstract] OR "systematic review"[title/abstract] OR review[title] OR "meta-analysis" [Publication Type] OR review [Publication Type] OR "systematic"[sb] OR scoping[title/abstract] OR "meta-synthesis"[title/abstract] OR "narrative review" [title/abstract] OR "critical review"[title/abstract] OR "critical synthesis"[title/abstract] OR "integrative review" [title/abstract] OR "integrative synthesis"[title/abstract] OR "qualitative review"[title/abstract] OR "metastudy"[title/abstract] OR "realist review"[title/abstract] OR "rapid review"[title/abstract] OR "umbrella review" [title/abstract] OR "BEME" [title/abstract] OR “consensus conference”[title/abstract] OR "medline" [title/abstract] OR "cinahl"[title/abstract] OR "PubMed"[title/abstract] OR "embase"[title/abstract] OR "psycInfo"[title/abstract]) AND ("Acad Med"[journal] OR "Adv Health Sci Educ Theory Pract"[journal] OR "Adv Med Educ Pract"[journal] OR "BMC Med Educ"[journal] OR "Can Med Educ J"[journal] OR “Clin Teach"[journal] OR "J Contin Educ Health Prof"[journal] OR "Teach Learn Med"[journal] OR "Perspect Med Educ"[journal] OR "Med Educ"[journal] OR "Med Educ Online"[journal] OR "Med Teach"[journal] OR "J Grad Med Educ"[journal] OR "Int J Med Educ"[journal])

**PubMed Search String for All Citations (LIMIT: 1999/01/01 – 2019/12/31)**

("Acad Med"[journal] OR "Adv Health Sci Educ Theory Pract"[journal] OR "Adv Med Educ Pract"[journal] OR "BMC Med Educ"[journal] OR "Can Med Educ J"[journal] OR “Clin Teach"[journal] OR "J Contin Educ Health Prof"[journal] OR "Teach Learn Med"[journal] OR "Perspect Med Educ"[journal] OR "Med Educ"[journal] OR "Med Educ Online"[journal] OR "Med Teach"[journal] OR "J Grad Med Educ"[journal] OR "Int J Med Educ"[journal])

**Web of Science (LIMIT: 1999/01/01 – 2019/12/31)**

((TI=("knowledge synthesis") OR AB=("knowledge synthesis")) OR (TI=("evidence synthesis") OR AB=("evidence synthesis")) OR (TI=("literature review") OR AB=("literature review")) OR

(TI=("evidence synthesis") OR AB=("evidence synthesis")) OR (TI=("systematic review") OR AB=("systematic review") OR (TI=”(review) OR AB=(review)) OR (DT=(review)) OR

(TI=(scoping) OR AB=(scoping)) OR (TI=("meta-synthesis") OR AB=("meta-synthesis")) OR

(TI=("narrative review") OR AB=("narrative review")) OR (TI=("critical review") OR AB=("critical review")) OR (TI=("critical synthesis") OR AB=(""critical synthesis")) OR (TI=("integrative review") OR AB=("integrative review")) OR (TI=("integrative synthesis") OR AB=("integrative synthesis")) OR (TI=("qualitative review") OR AB=("qualitative review")) OR (TI=("metastudy") OR AB=("metastudy")) OR (TI=("realist review") OR AB=("realist review")) OR (TI=("critical review") OR AB=("critical review")) OR (TI=("critical review") OR AB=("critical review")) OR (TI=("rapid review") OR AB=("rapid review")) OR (TI=("umbrella review") OR AB=("umbrella review")) OR (TI=("BEME") OR AB=("BEME")) OR (TI=(“consensus conference”) OR AB=(“consensus conference”)) OR (TI=("medline") OR AB=("medline")) OR (TI=("cinahl") OR AB=("cinahl")) OR (TI=("pubmed") OR AB=("pubmed")) OR (TI=("embase") OR AB=("embase")) OR (TI=("psychinfo") OR AB=("psychinfo"))) AND SO=((Advances in Health Sciences Education) OR (Clinical Teacher) OR (Teaching "and" Learning in Medicine) OR (Medical Education Online) OR (Medical Teacher))
